# PedWiz: a web-based tool for pedigree informatics

**DOI:** 10.3389/fgene.2013.00189

**Published:** 2013-09-25

**Authors:** Yeunjoo E. Song, Robert C. Elston

**Affiliations:** Department of Epidemiology and Biostatistics, Case Western Reserve UniversityCleveland, OH, USA

**Keywords:** pedigree, informatics, genetic similarity, identity-by-descent, relative pairs, family data

## Abstract

A novel web-based tool PedWiz that pipelines the informatics process for pedigree data is introduced. PedWiz is designed to assist researchers in the analysis of pedigree data. It provides a convenient tool for pedigree informatics: descriptive statistics, relative pairs, genetic similarity coefficients, the variance-covariance matrix for three estimated coefficients of allele identical-by-descent sharing as well as mean allele sharing, a plot of the pedigree structures, and a visualization of the identity coefficients. With a renewed interest in linkage and other family based methods, PedWiz will be a valuable tool for the analysis of family data.

## INTRODUCTION

When a researcher has collected or is provided with a set of nuclear family or extended pedigree data for genetic analysis, the first thing that needs to be done is to find out what information is available on the family or families before proceeding in the analysis of phenotype and/or genotype data to study the characteristics of a certain disease or trait, i.e., pedigree informatics. This can include descriptive statistics, visualization of family data, the degree of genetic relatedness among members of a family, and so on.

Descriptive statistics summarize and provide basic information on the family data, as done in the PEDINFO program in S.A.G.E. (2012). The visualization of family data is a fundamental task for both family studies and genetic counseling. There are many computer programs available that provide the graphical representation of pedigree data, including the R packages *kinship* (Zhao, 2006) and *pedantics* (Morrissey, 2010). The concept of genetic relatedness is essential in modern genetic analysis, and the applications of kinship and condensed identity coefficients are everywhere in analyses that have a genetic component. In human genetics, they are used in genotype prediction, calculation of genetic risk ratios for binary disease status, calculations of correlations between relatives, and robust linkage analysis. Robust linkage analysis, a powerful approach to map disease genes, is based on comparing the genetic marker profiles, i.e., allele identical-by-descent (IBD) sharing, of pairs of relatives. There are many software programs that calculate kinship and inbreeding coefficients, but not many for the nine condensed coefficients of IBD sharing.

A brief survey of available R packages with their relevant components of pedigree informatics is shown in **Table 1**. As can be seen, there is no program that provides all the different genetic similarity measurements together with the variance-covariance matrix of the estimated coefficients of IBD. Abney (2009)’s graphical algorithm for the computation of the generalized kinship coefficients is implemented in *idcoefs2* (written in C++, and implemented as the R package *identity*), and this is the only currently available program that outputs the nine condensed coefficients of IBD. The R package *ibdreg* by Schaid et al. (2007) has two functions, *sim.ibd.var* and *exact.ibd.var*, to calculate the variance-covariance of mean allele sharing, but not the variance-covariance of the individual coefficients of IBD. An essential part of score tests is the choice of the denominator variance, and some of these tests for genetic linkage require the variance-covariance of allele IBD sharing statistics under the null, i.e., of the coefficients of IBD. It would be useful to make available the variance-covariance matrix of these coefficients for a pedigree independent of the choice of test statistics, so that it can be used for different choices of test statistics. Currently, no such tools are available.

**Table 1:** R packages available for pedigree informatics.

Name	Plot	Stat	*F*	Φ	Δ	VC(2Φ)	VC(Δ)	Simulation
*adegenet*			√					
*gap*	√			√				
*geneland*			√					
*ibdreg*						√		
*identity*					√			
*kinship*	√			√				
*pedantics*	√	√	√					√
*pedigree*			√					
*pedigreemm*			√					
*GeneticsPed*			√					

*Plot, pedigree plot; stat, descriptive statistics; *F*, inbreeding coefficient; Φ, kinship coefficient; Δ, 9 condensed IBD coefficients; VC(2Φ), variance-covariance matrix of mean allele sharing; VC(Δ), variance-covariance matrix of 3 IBD coefficients.*

PedWiz (**Ped**igree Informatics **Wiz**ard) is designed to fulfill this need as a web-based tool for pedigree informatics, to assist researchers in the analysis of pedigree data. It provides a convenient “one-stop-shop” for pedigree informatics. It provides all the genetic similarity coefficients mentioned above, including the nine condensed coefficients of IBD and the variance-covariance matrix of the one-locus three marginal coefficients of allele IBD sharing, as well as other pedigree descriptive statistics. Additionally, it provides a plot of the pedigree structure and a visualization of the identity coefficients, something that no other program provides. PedWiz is an automated pipeline for extracting pedigree informatics before conducting specialized analyses of phenotype and/or genotype data.

## MATERIAL AND METHODS

### IMPLEMENTATION

The web interface of PedWiz is implemented using a combination of XHTML (eXtensible HyperText Markup Language), CSS (Cascading Style Sheets), and PHP (Hypertext Preprocessor) on an Apache web server. The interactivity is provided by JavaScript and Ajax technologies. Custom Python modules handle the overall flow of the pipeline by calling pre-existing programs written in C++ or R.

### USER INPUT

PedWiz accepts a plain ASCII text file format for pedigree input. Since PedWiz extracts the information contained in a pedigree structure, it requires a pedigree file to have five essential columns: pedigree ID, individual ID, the two parents’ IDs and sex. These five columns do not need be in any specific order, nor need they be consecutive. If a pedigree file contains other columns, they are ignored. The pedigree file is required to be in either tab-delimited or comma-delimited format. It may optionally contain a header line specifying the names of the columns. The user inputs configuration information and the location of the pedigree file through a user-friendly interface, and then submits it to start the analysis pipeline.

### ANALYSIS TOOLS

**FIGURE 1:**
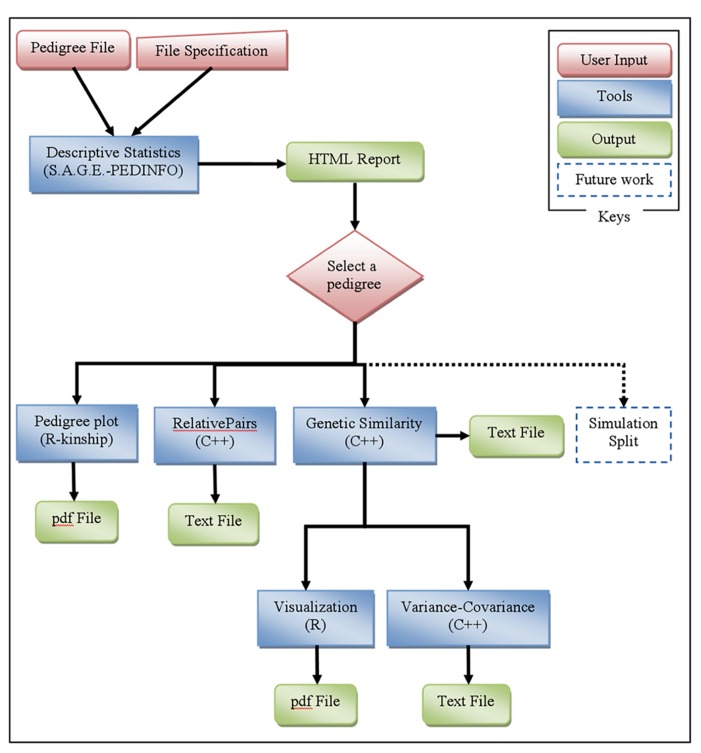
**PedWiz overview.** This figure illustrates the analysis pipeline implemented in PedWiz. It consists of six tools to mine the information in a pedigree structure: descriptive statistics, pedigree plot, relative pairs, genetic similarity coefficients, visualization of genetic similarity coefficients, and the variance-covariance matrix of coefficients of IBD. The tools denoted by dotted lines are anticipated future extensions.

Once the user submits a pedigree file and configuration information, the informatics process starts by running the first tool. Currently, the PedWiz process consists of six main tools (**Figure 1**). The complete process utilizes many internal Python scripts (which are not detailed here) to create junctions between the programs (format compatibility) and to create the necessary R scripts.

#### The descriptive statistics tool

This tool is used to calculate the descriptive statistics for each pedigree contained in the user-submitted pedigree file. PedWiz utilizes the existing C++ program PEDINFO of the S.A.G.E. package (v6.3 with *each_pedigree = true* option). PEDINFO provides many useful descriptive statistics on pedigree data including means, standard deviations; family, sibship and pedigree sizes; and counts of each type of relative pair. The results are parsed and reported to the user by PedWiz as a table on the website. From here, the user selects a pedigree to proceed with other tools.

#### The pedigree plot tool

This tool is used to visualize a pedigree. PedWiz utilizes the R package *kinship* to generate the plot (Zhao, 2005). As in a typical pedigree diagram, males and females are shown as squares and circles, respectively. The resulting pedigree plot is reported to the user as a pdf file on the website.

#### The relative pairs tool

This tool is used to report all relative pairs existing in a pedigree. PedWiz uses an internal C++ program that finds all existing relative pairs by traversing the pedigree structure recursively as done in the FCOR program in S.A.G.E. (2012). The results are reported to the user on the website as a text file containing the relative pair matrix and the list of relative pairs for each relative type.

#### The genetic similarity tool

This tool is used to provide the various genetic similarity coefficients. PedWiz uses an internal C++ program to perform this task. The results include two matrices; one is the matrix of kinship/inbreeding coefficients (inbreeding coefficients on the diagonal and kinship coefficients off the diagonal), and the other is the matrix of nine condensed coefficients of IBD. The coefficients of relationship, which are twice the kinship coefficients, can be easily calculated from the kinship/inbreeding coefficients. The resulting matrices are reported to the user on the website as a text file.

#### The visualization of genetic similarity tool

This tool is used to visualize the two matrices generated by the genetic similarity tool. PedWiz uses a custom R script to represent a matrix graphically as a heat map. The resulting heat maps are reported to the user as a pdf file on the website.

#### The variance-covariance of genetic similarity tool

This tool is used to find the variance-covariance matrix of the coefficients reported by the genetic similarity tool. PedWiz uses an internal C++ program to perform this task. The variance-covariance matrix of kinship coefficients is estimated by an exact method given by Chen and Abecasis (2006). The variance-covariance matrix of IBD coefficients is estimated by a simulation method, given a pedigree structure (MacCluer et al., 1986), based on 500 simulation replicates. The simulation method approximates the distribution of IBD states by gene dropping, so it can be used regardless of pedigree size and structure. The results are reported to the user on the website as a text file.

## RESULTS

**FIGURE 2:**
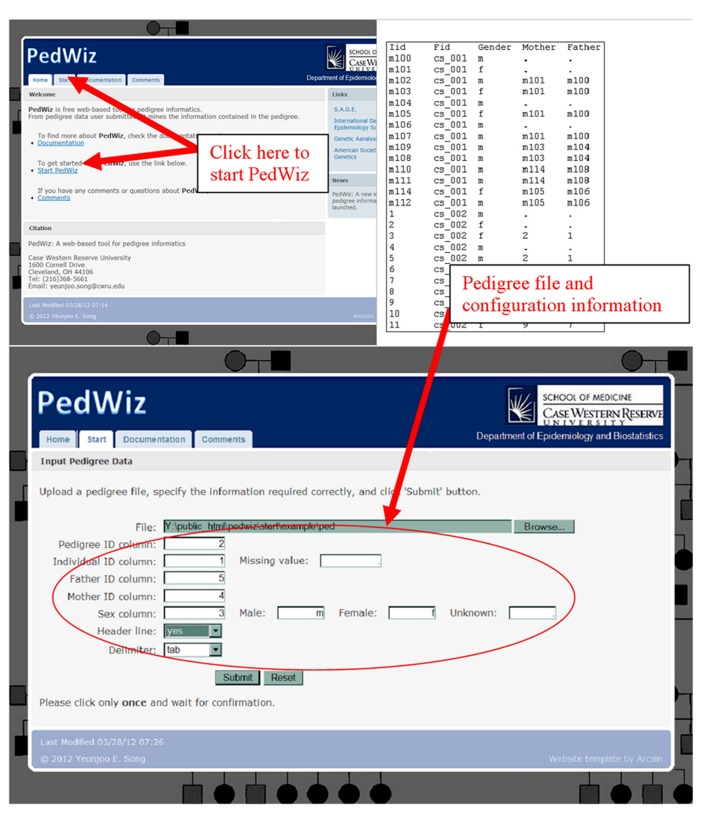
**Starting PedWiz.** This figure illustrates the user interface to start PedWiz.

We developed a novel web-based tool that pipelines the informatics process for pedigree data. PedWiz may be accessed at . Here we present an application example using pedigree data from the Madeline 2.0 website (Trager et al., 2007). These pedigree data contain a consanguineous marriage between cousins. The user inputs configuration information and the location of the pedigree file through the interface on the website as shown in **Figure 2**.

**FIGURE 3:**
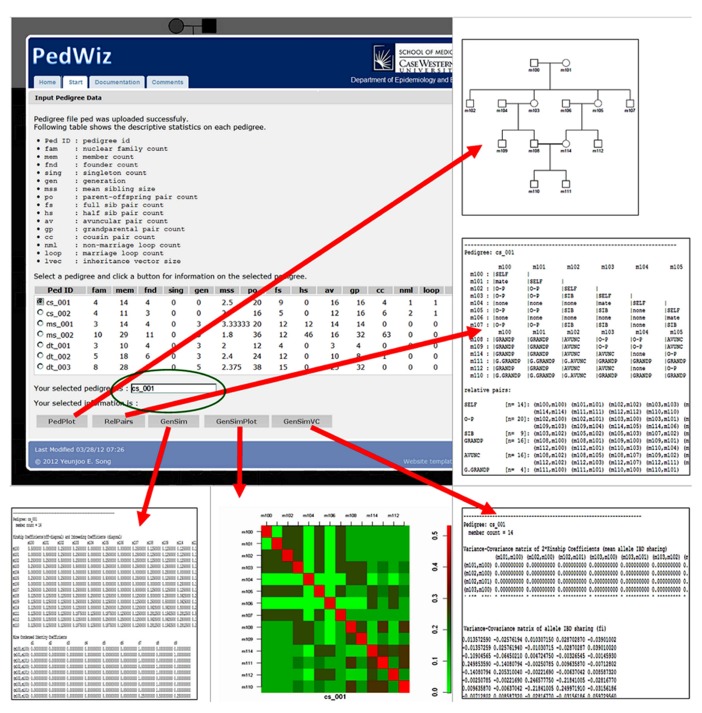
**Different types of outputs from PedWiz.** All results are accessed through a set of buttons under the descriptive statistics table for each pedigree.

After configuration information and the location of the pedigree file have been submitted by the user, PedWiz produces a table with the descriptive statistics for each pedigree on the website as shown in **Figure 3**. All results are accessed through a set of buttons under the descriptive statistics table for each pedigree. The user uses a radio button to select a pedigree for an analysis pipeline. This selection information is reflected under the table (shown in the green eclipse). The resulting output from each tool for the example pedigree is shown also.

## DISCUSSION

We developed a novel web-based tool PedWiz that pipelines the informatics process for pedigree data. PedWiz is designed to assist researchers in the analysis of pedigree data. It provides a convenient tool for pedigree informatics: descriptive statistics, relative pairs, genetic similarity coefficients, the variance-covariance matrix of three coefficients of allele IBD sharing, as well as mean allele sharing, a plot of the pedigree structure, and visualization of identity coefficients. PedWiz is an automated pipeline for extracting pedigree informatics before conducting specialized analysis of phenotype and/or genotype data.

Emerging availability of whole genome sequence data has led to a renewed interest in linkage and other family based methods (Ott et al., 2011). Many researchers have been emphasizing the importance and advantages of family studies all along (Clerget-Darpoux and Elston, 2007; Stein and Elston, 2009), especially to interpret next generation sequence data (Bailey-Wilson and Wilson, 2011; Wijsman, 2012). Family study designs provide not only the enrichment of genetic loci containing rare variants, but also methods to control for genetic heterogeneity and population stratification. PedWiz is a valuable tool for initial analysis of those family data.

Additionally, the results from each tool in Pedwiz will be useful for later analysis of phenotype and/or genotype data. As stated before, an essential part of score tests is the choice of the denominator variance, and some of these tests for genetic linkage require the variance-covariance of the coefficients of IBD. No software tools are currently available to provide this information independent of the choice of test statistics. The variance-covariance of the genetic similarity tool of PedWiz provides this need, so that it can be used for different choices of test statistics. The information from the genetic similarity tool of PedWiz can be used for weighting pedigrees of different sizes. Another potential use of this tool is for selecting families with the most information in terms of genetic relatedness that would best suit a phenotype/genotype analysis of choice. Selecting families with multiple affected subjects, or families with extreme values, is known to provide improved ability to measure, and detect, the effects of rare variants (Ionita-Laza and Ottman, 2011; Wijsman, 2012). The strategy of selecting “large linked families” for initial screening has long been a successful strategy (Bowden et al., 2010). To be successful with this approach, selecting families with a real linkage signal in specific regions is essential. This new tool will be useful for selecting such families when used together with phenotype/genotype information.

With a modular design, each analysis tool within PedWiz is independent of the others, so it is very easy to extend and add more tools. Planned additions in the near future are simulation and pedigree split tools, shown in **Figure 1** with dotted lines. PedWiz is currently specialized to deal with the information contained within pedigree structures only. Therefore, it is very fast and safe with regard to data transfer over the web. However, it is always possible to add more pipeline modules that could process the information from phenotype and/or genotype data. Good candidates for this addition would be simulation conditional on given phenotype and/or genotype data, and imputation. Another extension that could be added on is the inclusion of a backend database to save data and results for reuse.

The genetic similarity tool of PedWiz is specifically designed to provide the information on within-pedigree relatedness. As a reviewr pointed out, a tool that addresses between-pedigree relatedness, especially for pedigrees from a relatively isolated population like the Hutterites, would be a useful addition to PedWiz. Cryptic relatedness among unrelated individuals can be estimated by incorporating a number of dense markers across different chromosomes (Weir et al., 2006; Bink et al., 2008; Astle and Balding, 2009; Sillanpää, 2011). There are many software tools available to estimate the genome-average relatedness, for example, SPAGeDi (Hardy and Vekemans, 2002), PLINK (Purcell et al., 2007), FEST (Skare et al., 2009), CoCoa (Maenhout et al., 2009), CrypticIBDcheck (Nembot-Simo et al., 2013). Adding this to PedWiz would require an extension to process information from phenotype and/or genotype data, as mentioned above.

## Conflict of Interest Statement

The authors declare that the research was conducted in the absence of any commercial or financial relationships that could be construed as a potential conflict of interest.
